# Believing in the Powers of Mindfulness: A Thematic Narrative Approach and the Development of a New Scale

**DOI:** 10.1007/s12671-023-02164-x

**Published:** 2023-06-21

**Authors:** Masoumeh Rahmani, Valerie van Mulukom, Miguel Farias

**Affiliations:** 1 School of Social and Cultural Studies, Victoria University of Wellington, Wellington, New Zealand; 2 Brain, Belief and Behaviour Lab, Centre for Trust, Peace, and Social Relations, Coventry University, Coventry, UK

**Keywords:** Mindfulness, Mixed methods, Narrative analysis, Beliefs, Scale development

## Abstract

**Objectives:**

The beliefs and expectations people bring into mindfulness practice can affect the measurement outcomes of interventions. The aim of this mixed-method study was to examine the key beliefs in the powers of mindfulness—understood as non-judgmental awareness of the present moment—to transform the individual and the society, and to develop and validate the Belief in the Powers of Mindfulness Scale (BPMS).

**Method:**

In-depth, semi-structured interviews were conducted with mindfulness meditators (*n* = 32), including follow-up interviews (*n* = 22). Qualitative data were analysed through a thematic narrative approach. Participants (*n* = 458) completed a questionnaire that included the new scale.

**Results:**

Participants’ key beliefs were thematically analysed in three transformation themes: interpersonal relationships and compassion, peace and violence, and the inner world—themes were encapsulated in the BPMS. Ideas presented in each theme were undergirded by a host of ideologies, epistemic claims, and metaphysical assumptions about the nature of mind, self, and reality—which are predicated by broader cultural trends such as expressive individualism, perennial philosophy, and New Age sentiments and ideals. The BPMS showed strong internal consistency and convergent validity, and individuals who were older and more spiritual practised mindfulness more often and for longer, and self-reported greater mindfulness skills, and scored higher on the BPMS.

**Conclusions:**

Findings illustrate the persisting importance of attending to people’s beliefs and expectations in mindfulness interventions and further the call for a contextual approach that accounts for cultural factors. The newly developed BPMS may assist with the measuring of peoples’ beliefs and expectations.

Mindfulness meditation, in its contemporary usage, refers to a range of formal and informal practices that involve “paying attention on purpose, in the present moment, and non-judgementally to the unfolding of experience moment by moment” (Kabat-Zinn, 2003, p. 145). Mindfulness meditation has become a widely used technique for achieving well-being and personal change. There are thousands of available books and courses, and hundreds of apps, as well as a variety of products aimed at transforming unique aspects of our life ranging from weight loss through mindful eating to better sleep, or even attaining a more successful career. The popularity of mindfulness is in part connected to its appeal to individualism and the culture of self-improvement (Payne, 2016), and the increase in consumer demand for alternative healing practices, accompanied by the proliferation of popular publications on health information and self-improvement (Barker, 2014).

The popular literature on mindfulness builds upon a growing academic interest, with mindfulness research showing exponential growth in the past two decades (Baminiwatta & Solangaarachchi, 2021). The academic exploration of the benefits of this technique now extends well beyond physical or psychological well-being for which there is considerable evidence (Creswell, 2017; Goyal & Rusch, 2021). The past decade has witnessed new research on its interpersonal benefits, such as an increase in empathy and compassion (Block‐Lerner et al., 2007; Feldman & Kuyken, 2011; Salzberg, 2011), cultivation of prosocial behaviour (Berry et al., 2018; Donald et al., 2019; Hafenbrack et al., 2020), an enhancement of interpersonal relationships (Bihari & Mullan, 2014; Moll et al., 2015), reductions in aggression and violence (Gillions et al., 2019), and a decrease in implicit age and race biases or the automatic processes leading to prejudiced behaviour (Lueke & Gibson, 2015). Although recent scholarship has come to question some of these claims (Kreplin et al., 2018; Poulin et al., 2021; Ridderinkhof et al., 2017), the abovementioned positive associations and assumptions about the powers of mindfulness continue to capture researchers and the public’s imagination.

The plethora of academic and popular literature indicates that people hold a certain set of *beliefs* about the powers of mindfulness. The wide-ranging applications of the potential transformative powers of mindfulness at the individual, interpersonal, and even societal level, including the promotion of its use in schools, show a genuine enthusiasm by individuals and institutions. Such enthusiasm about the transformational power of mindfulness is vividly expressed in Kabat-Zinn’s preface to *Mindful Nation UK* (2015, p. 10), the report of the British Mindfulness All-Party Parliamentary Group. He stated that mindfulness has the potential to transform “who we are as human beings, and individual citizens, as communities and societies, as nations, and as a species.”

Definitions and conceptions of mindfulness vary considerably (Hitchcock et al., 2016; Lester et al., 2018). Research suggests peoples’ reasons for starting mindfulness meditation and continuing the practice can be very heterogeneous (Pepping et al., 2016; Sedlmeier & Theumer, 2020)—ranging from the means to deal with psychological and physical problems (stress, anxiety, panic, and depression), to a tool for the enrichment of one’s life or spiritual growth. Studies on meditation in general also suggest that peoples’ motivations can change with increased practice (Jiwani et al., 2022; Schmidt, 2013; Sedlmeier & Theumer, 2020; Shapiro, 1992) and that increased practice and socialisation into meditation groups can also lead to changes in self-concept, language, and worldviews (Rahmani, 2022). As the variation in beliefs of the power of mindfulness is typically unassessed in interventions, they are likely to go unnoticed. This may have important consequences for researchers, clinicians, and the meditators alike, as varying sets of beliefs about mindfulness may affect the outcomes of meditation (Landau et al., 2022; Lester & Murrell, 2019), for example, through different expectations (Farb, 2012; Greenberg et al., 2006). Yet, beliefs and expectations are often unaccounted in mindfulness studies and only a few studies control for the effects of expectations (Creswell et al., 2014; Haddad et al., 2020; Ribeiro et al., 2018).

What might such beliefs in the powers of mindfulness meditation look like? Here, we start by sketching the social–historical context in which modern mindfulness arose and discussing the Western cultural ideas in which it is embedded, in particular as attached to the social movement known as New Age spirituality.

In a general sense, mindfulness is often thought to entail intention, attention, and awareness typically following Jon Kabat-Zinn’s famous definition of mindfulness (1994)—as “paying attention in a particular way, on purpose, in the present moment, and non-judgmentally.” In a specific sense, the term originates from the Pali term *sati* (Sanskrit: *smrti*), which originally meant “to remember”, “to recollect”, and “to bear in mind”. As Sharf (2015, p. 473) has noted sati, in the context of Satipaṭṭhāna Sutta (the section of the Pali Canon that discusses mindfulness), involves “bearing in mind the virtuous dharmas so as to properly apprehend, from moment to moment, the true nature of phenomena.” The cultivation of mindfulness, as delineated in classical Theravada Buddhist texts, is therefore a continuous strive to bear in mind the body, feeling, mental states, and mental factors, all of which require attention, memory, and metacognition, as well as conceptual understanding (Thompson, 2017).

In a traditional Buddhist context, meditation is not practised in isolation from ethical, doctrinal, and liturgical commitments (King, 2019). Historically, Buddhist meditation techniques were solely accessible to ordained monks and took aim at enlightenment by cultivating insight into the true nature of self and reality (Gombrich, 2006; Gombrich & Obeyesekere, 1988); the transformative powers of meditation are nestled in the tradition’s social, existential, ethical, soteriological, and cosmological order (King, 2019; Sharf, 2015). Modern therapeutic adaptations of mindfulness meditation, however, are often presented as a freestanding technique, often decontextualised from religious concerns (McMahan & Braun, 2017; Rahmani, 2020). Meditation in the Buddhist context aims for liberation from the realms of rebirth, whereas modern mindfulness aims for psychological and physiological wellness. Another way of contrasting these differing purposes is that within a Buddhist framework one cultivates the realisation that existence is fundamentally characterised by suffering/unsatisfactoriness, and seeks to transcend it, whereas in the modern therapeutic application of mindfulness the aim is to better cope with existence and to foster well-being and self-enhancement (Bodhi, 2016; Lewis & Rozelle, 2016).

These transformations in the purpose and significance of mindfulness meditation did not occur overnight. Much has been written on the historical, political, and sociocultural processes contributing to the transformation of Buddhist meditation and its integration in Western contexts (Carrette & King, 2004; McMahan, 2008). Historically, they are born out of the colonial encounter between Europe and Asia, which led to the emergence of Buddhist modernism—a reform movement led by elite Theravada Buddhist monks who sought to revive their religion’s loss of power, prestige, and legitimacy in the wake of colonisation and Christianisation (McMahan, 2004).

During this period of reform and revival, popular and accessible books on Buddhist philosophy and meditational practices went into print; urban meditation centres opened to the public; meditation became widely accessible, and enlightenment became a possibility in this lifetime (Braun, 2013). Importantly, interpretations of the “true” or “pure” Buddhism emerged as a supposedly rational and humanistic philosophy devoid of dogma, or rituals, the Buddha was portrayed as a moral philosopher, and Buddhist meditation became “a science of the mind” (Lopez, 2012, p. 11). This transformed Buddhism placed an emphasis on meditative experience, shifted religious authority and responsibility of salvation to the individual, and adopted a new universalist approach and an international outlook (Sharf, 2015). Conceptually, by reshaping Buddhism in the light of Western notions of science, democracy, individualism, and enlightenment, Buddhist modernism was responding to the western and Christian stereotypical view of Asian religions as “primitive”, “superstitious”, and “mystical” (King, 2019; McMahan, 2004).

The trends established by Buddhist modernism continue to shape the public’s imagination of Buddhism and Buddhist meditation in the West. Above all, the representations of Buddhism as a tradition uniquely compatible with modern science played an essential role in the successful transmission of Buddhist meditation to the West, forming the intellectual foundation for the emergence of the mindfulness-based programmes that are popular today (King, 2019; McMahan, 2004). It is important to note that similar processes of decontextualisation and “scientisation” occurred with other Eastern traditions that travelled West, such as with Transcendental Meditation (Farias & Rahmani, 2020). As Kabat-Zinn (2011: p. 282) himself notes, in order to facilitate the introduction of mindfulness to Western mainstream culture and “make it acceptable” to non-Buddhists, he “bent over backward” to select a language that “avoided as much as possible the risk of it being seen as Buddhist, ‘New Age’ ‘Eastern Mysticism’ or just plain ‘flakey.’”.

One of the key beliefs of this social movement was that techniques taken from spiritual traditions, like meditation and yoga, could be stripped of their ritual and doctrinal contexts and be used to quickly instigate deep personal transformation (Heelas,^1996^). This has led to a growing discussion on whether to use more explicitly Buddhist ideas in mindfulness practice, which has given rise to the so-called second-generation mindfulness-based interventions, that attempt to be more overtly spiritual (Van Gordon & Shonin, 2020). Although much of the current mindfulness literature is focused on personal well-being, there is nevertheless a very broad spectrum of possible benefits within the scientific literature, not the least the possibility of developing through meditation a state of “bare” or “pure awareness”.

The translation of mindfulness as “bare attention” by Buddhist modernists was first popularised in a best-selling book by the German Theravada monk, Nyanaponika Thera, and has become the common one employed by mindfulness researchers today, along with that of “pure awareness” (Kabat-Zinn, 2015). That the etymology of mindfulness is being somewhat inaccurately presented is less important than what this change in meaning reveals—a portrayal of mindfulness as something tremendously powerful and with deep roots in the Western imagination; seeing things as they *really* are, free from the shackles of our habitual perception and our formal learning. This has obvious resonances with Romantic philosophical ideas on the quest to self-discovery which involves moving away from “the narrow chinks of [one’s] cavern and cleansing the doors of our perception” (Blake, 1975 [1794], p. xxii). This view of mindfulness as an introspective technology is at home with Romantic sensibilities including *expressive individualism*—what Charles Taylor calls the culture of authenticity—which is the product of the subjective turn and its displacement of the moral accent: whereas in earlier theistic moral views, morality was sought from a source that transcended human beings (God; religion), it is now something to be found in the inner depths of ourselves (Taylor, 1992, 2007). It also echoes the New Age privatisation of Asian wisdom and more generally the relocation of religious to the private sphere of individual choice (Carrette & King, 2004). The historical continuity between Romantic ideas and New Age spirituality is well established (Hanegraaff, 1996), and may well help in part to explain the contemporary appeal of mindfulness.

The variety of perspectives of what mindfulness might entail is mirrored in the concepts of mindfulness held by laypeople. Only a few studies have been conducted so far to examine laypeople’s ideas of mindfulness; they show that non-experts hold heterogeneous conceptions of mindfulness (Alvear et al., 2022): some believe that mindfulness is a technique that is meant to increase emotional control (Hitchcock et al., 2016), to improve prosocial behaviour and emotional positivity (Kaplan et al., 2018), or to give one a competitive edge in the business world (Van Doren et al., 2022), or that it is a relaxation or a strictly religious practice (Lester et al., 2018). Furthermore, there remains a lack of consensus within the scientific community over the exact definition of mindfulness or its underlying facets (Van Dam et al., 2018). There are more than a dozen of different scales that claim to measure mindfulness, each offering its own conceptualisation of the construct as well as a different combination of its sub-components (Ackerman, 2021; Medvedev et al., 2022). These conceptual disagreements are probably a reflection of the various perspectives espoused by researchers, ranging from a cognitive account of mindfulness as a particular form of paying attention to the more Romantic notion of this technique as a doorway to experiencing pure awareness and bringing about societal change.

In the present article, we examined some of the key beliefs about the power of mindfulness to transform the individual and society through a mixed-methods approach, using interviews and the development of a new scale, the Belief in the Powers of Mindfulness Scale (BPMS). Note that we were interested in *assessing* people’s beliefs about the powers of mindfulness, not in evaluating the validity of such claims (for recent comprehensive reviews of the literature, see Farias et al., 2021). This study adopted a semi-sequential, mixed-method design (Creswell & Creswell, 2017; Plano Clark & Ivankova, 2016), intersecting ethnographic fieldwork within our qualitative and quantitative mixed-method approach. The initial qualitative phase involved literature review and ethnographic fieldwork followed by semi-structured, in-depth interviews with 10 participants. Insights from fieldwork and the thematic analysis of the interviews were integrated to create the BPMS items. Thereafter, data collection including the additional in-depth interviews (*n* = 22) took place concurrent with the survey (*n* = 458). The final stage of data collection involved follow-up interviews (*n* = 22).

In short, there are reasons to suggest that individuals practising mindfulness may hold a variety of beliefs about its powers to promote personal change. Here, we sought to examine these beliefs through a mixed-method approach including longitudinal interviews (Study 1) and the development of a new scale on beliefs in the powers of mindfulness (Study 2).

## Study 1

Study 1 comprised the qualitative assessment of common beliefs in the transformative power of mindfulness. Where do these beliefs stem from and what assumptions, if any, undergird them? How are these beliefs conceptually related to one another and/or to broader cultural trends? To address these questions, Study 1 utilised in-depth interviews with mindfulness meditators and teachers.

### Method

#### Participants

Interview participants were recruited through a combination of recruitment strategies: in person during Rahmani’s fieldwork in the UK between 2017 and 2018 (Workplace Masterclass Programme and an 8-week Mindfulness-Based Cognitive Therapy, MBCT (Segal et al., 2013) course at the Oxford Mindfulness Centre; Mindful Living Show, and Mindfulness in School Project Conference), snowball sampling, and advertisement via Facebook and Twitter. Because we wanted to examine the concept of mindfulness as presented in the West—a non-religious practice—we restricted our recruitment to individuals that perceived themselves to be non-religious. Thus, our recruitment statement read: “The purpose of this research is to explore what attracts non-religious individuals to mindfulness meditation and how the practice influences their understanding of themselves and the world”.

Eighteen women and 14 men between the ages of 19 and 69 were interviewed. Sixteen participants were from the USA and 16 were from the UK (although 3 of these individuals resided outside of the UK at the time of the interview). Most (70%) had university education; occupation ranged from retired or unemployed (15%), educators (36%), software developers (9%), marketing consultants (9%), and students (6%), with remaining individuals distributed among other occupations (25%). Nineteen were born in Christian households, nine were raised in secular families, three had mixed-faith parents, and one participant was raised in the Hindu tradition.

The participants had different levels of engagement with mindfulness meditation (at the time of the initial interview, the length of their engagement with the practised ranged from 1 participant 0–6 months, 10 participants 6–12 months, 7 participants 2–5 years, 9 participants 5–10 years, and 5 participants over 10 years). Half of the participants (sixteen) were engaged with “institutionalised” mindfulness (they did courses on MBCT or Mindfulness-Based Stress Reduction, MBSR (Kabat-Zinn, 2013) and the other half practised “unstructured” mindfulness (learned via Apps, CDs, in therapy, or yoga classes).

#### Procedure

This qualitative material is based on in-depth, semi-structured interviews with 32 mindfulness meditators/teachers, including 22 longitudinal case studies (a total of 54 interviews, resulting in 60 hr of audio-recordings; each interview was approximately 60 min long). All interviews were conducted through videoconferencing. The follow-up interviews were conducted 8 to 12 months after the initial ones and were individually designed to examine the influence of mindfulness on the participants’ language and worldview. The interviews were subsequently transcribed and analysed using a combination of thematic and structural narrative analyses (Riessman, 1993). Consent forms were signed prior to the interviews and pseudonyms were used to ensure the anonymity of all participants.

#### Measures

The interviews were semi-structured, steered to some extent by an interview guide that addressed themes such as participants’ background, religious or non-religious upbringing, their introduction to mindfulness, and their experience and understanding of the practice and examples of its application in their day-to-day lives. The interviews also included conceptual questions such as the participants’ definition of mindfulness, religion, and reality along with a set of provocative issues such as the implementation of mindfulness in the military. Moreover, all interview questions were open-ended in design, allowing for participants to build up their narratives at their own pace and to discuss matters they considered important. The present study was part of a larger mixed-method project that examined (1) the ways in which mindfulness meditation is presented as a non-religious enterprise and thereby appeals to the atheists and non-religious, and (2) the influence of the practice on the practitioners’ worldviews. Therefore, several interview questions also focused on unpacking what non-religious identities meant to the participants.

#### Data Analyses

The qualitative dimension of this research is grounded in an interpretivist framework. In this research, interpretations were made in relation to the aspects of the wider sociocultural and historical realities that shape and influence the participant, the phenomenon, and the participant’s understanding of the phenomenon. The qualitative material was analysed through a dynamic combination of thematic and structural narrative analyses. Structural narrative analysis mainly concerns itself with context—rather than content—and aims for extracting meanings encoded in the form of the narrative, by constantly posing questions as to: how is the narrative organised and why is it developed in this particular way? (Riessman, 1993). Thematic narrative analysis was therefore incorporated to organise and analyse the data in terms of their content: what is said, rather than how or why it is said. Thematic analysis of narratives, as opposed to structural, is somewhat similar to grounded theory’s open coding methods, albeit the former preserves sequence, rather than thematically coding segments. The thematic arrangement of the data was done using NVivo10, while structural analysis and treatment of the material were done manually. Some examples of the thematic codes include attitudes towards parental religion; first encounter with mindfulness; interpersonal relationships; assumptions about mindfulness; assumptions about human nature; conceptions of ultimate reality; moral dispositions; career trajectories; doubts and conflicts; goals; common rhetoric; and definitions of mindfulness and religion. An example of structural analysis includes examining the context in which particular rhetorical strategies were operationalised to resolve doubt or ambivalence (Rahmani, 2020).

### Results

Participants shared a range of ideas and dispositions about the transformative efficacy of mindfulness meditation. In what follows, we detail the key transformation themes, including (1) interpersonal relationships and compassion, (2) peace and violence, and (3) the inner world.

#### Interpersonal Relationships and Compassion

In describing the effects of mindfulness on their interpersonal relationships, participants presented narratives and attitudes that rested on a continuum between (a) becoming an engaged listener and (b) a detached, disinterested observer. For instance, some emphasised that mindfulness enabled them to let go of their desire to control situations; to reduce their impulse to “jump in to help or fix things for others”; to become better listeners and more “willing to learn others’ perspective”; and to manage conflict with others by recognising their own anger.

Lilith [40s; UK]: When I look at my relationship with my mom for example, that’s really improved in recent years as my practice has really become very regular. Yeah, I’m not involved in any past stories anymore. I just [pause] I enjoy some quality relationship with her. Yeah, and also, I don’t force myself to be this perfect daughter. So, I am kind to myself with the situation. I give what I can, but I also recognise when it starts to become uncomfortable for me. And so, I create the space that I need from her. Also, it [mindfulness] helped me recognise, and this is a powerful thing that happens with mindfulness, it made me realise that actually there’s two people. What’s their shit and what’s your shit and that’s a really powerful thing. I don't have to resolve her stuff.

In addition to this perspective, some of the participants strongly advocated for a detached and disinterested approach, particularly in the context of interpersonal conflicts. They argued that mindfulness helped them to emotionally detach from difficult situations, often by “walking away” from conflicts; that it helped them to “try not to get caught up in conflict or care about people’s opinions”; to not “waste energy” on others altogether; or simply control their exterior in emotionally taxing situations.

James [40s; UK]: The last couple of days before he [father] died, um my sister and my mom were with me, and we were walking out, and they were both in tears and I was quiet. [pause] I mean I was down. I felt sad but I wasn’t in the same sort of um emotional state. And my sister told me ‘I don’t know how you can, um how you can’t be um upset, you’re not crying.’ And I didn’t have an answer. But I think it’s because, I can choose thoughts […] with mindfulness I’ve found that I can control my or choose what I want to think about.

Despite this diversity, they all construed the behavioural changes in a positive light and saw them as favourable effects of their mindfulness practice. Moreover, the participants’ discussions about interpersonal relationships were replete with references to the concept of compassion. While all participants considered compassion as an integral facet of mindfulness and central to the ways in which the practice had influenced their relationships, here too, responses could be conceived on a continuum from those who emphasised self-compassion vs. those who emphasised compassion to others. Moreover, when participants were probed to reflect on the links between mindfulness and compassion, they often conceptualised compassion as an innate human quality, and mindfulness as a powerful tool for unleashing compassion within us:

Penelope [40s; UK]: I think there’s less of an effort needed [for me] to have compassion for more people.

Author: Doesn’t that come with maturity? And just basically living and having more [life] experience? Or are you associating that with mindfulness?

Penelope: I would say no immediately only because […] I must have those qualities inside of me. I believe that everybody has these qualities inside of them. But I think that mine do surface [pause] perhaps more than other people. So, perhaps, perhaps I would’ve been more compassionate at this age. Um but when I, if I compare myself to people who, who perhaps haven’t practised Mindfulness, there seems to be a difference.

For others, the theme of compassion was conceptually intertwined with the notions of “cosmic oneness”. In other words, the framework upon which some participants drew upon to theorise and cultivate compassion within themselves rested on a metaphysical view of monistic oneness:

Olivia [30s; UK]: I think for me, the most important idea, or what helps me get out of bed type of thing is knowing that I’m connected to everything around me and that everything is connected to me and I think it’s really interesting that the more I look at the similarities between how we function in a, in a physical sense and how the universe functions. There’re so many similarities between how we’re made up and that’s quite comforting to me to know that. And it allows me, I think, to be more compassionate with people.

While the above conceptual framings of compassion—as (1) an innate hidden human quality, or (2) a premised on cosmic oneness—were prevalent, there were a smaller group of participants who spoke of compassion as a skill that could be cultivated through mindfulness. Yet, irrespective of this difference, what all participants shared was the conviction that mindfulness has the power to generate compassion and thus positively impact the world.

#### Peace and Violence

Given the growth of literature on mindfulness and its potential to reduce violence and aggression in a variety of contexts (e.g. Gillions et al., 2019; Morley et al., 2019), we asked various questions to explore this topic, including one on the use of mindfulness in the military. The purpose of this question was twofold: to explore ideas about the power and efficacy of mindfulness in reducing violence and to gauge their position on the ethics of promoting such interventions.

Except for two participants, who found the application of mindfulness in the British and American military deeply problematic and conflicting with what they conceived as the Buddhist ethics of non-violence, the vast majority of the participants (30 out of 32) expressed their support for such interventions. They reasoned that mindfulness would help the soldiers to better cope with post-traumatic stress disorder (PTSD); that it might increase their focus and attention; and that the soldiers would be less likely to make impulsive decisions on the battlefield, because they will be “present and not a slave to [their] emotions or amygdala”.

Moreover, almost half of the participants (15 out of 32) seemed convinced that mindfulness will cultivate compassion and enable soldiers to “see the humanity in others” in such a way that they ought to “quit the army” and change their career.

Rhiannon [40s; US]: I think that’s great. I think that soldiers, like people who see combat, are on crazy amount of stress and a lot of times come back from you know Middle East or whatever with PTSD and from what I understand mindfulness is a great way to mitigate the effects of PTSD. So, I would vote for that […] I don’t think mindfulness makes soldiers into killing machines. I think it would turn them into happier more peaceful soldiers. I don’t know if this is true, but I read an article online about how happy cows give better milk [laughs] […] I’m definitely not cool with the whole history of British and American imperialism. I think that’s generally not a good idea. But I think also that there is value in dealing with what is. You can have a perfect picture in your mind of what the world should be. But we also have to deal with what is. And if there are human beings who struggle with anxiety and PTSD, who could be helped by mindfulness. And maybe if the soldiers do that, they will quit the military all together.

Similar to the theme of interpersonal relationships, the concept of compassion was integral to participants’ discussions about the power of mindfulness in promoting peace and positively impacting the world.

Marv [30s; US]: The inequities of the society um [pause] regionally, nationally, and globally, are still very, very real. And um, you know when I try to think about self-com[passion], lovingkindness and doing a metta practice and having those rays of positivity radiated out, I have to think like, ‘oh I have to think positively of Donald Trump and his supporters?’ Like that’s challenging. And I, I take solace in the fact that perhaps if they had the same mental peace, that composure that I believe mindfulness can help give people, and they also felt the same compassion that mindfulness can help instil in people, that those inequities would be less in our world.

That said, a few participants seemed ambivalent about the efficiency of mindfulness in cultivating ethics and compassion in and of itself, especially if taught and practised removed from an ethical framework.

Timothy [60s; US]: This question comes up: can you have effective mindfulness without the right livelihood, right thought, right speech, can you have one without the other? I would say, you can, but you can’t get very far. That you’d be stunted in your ethics. That meditation cannot be as fruitful and fulfilling […] but I can't imagine someone who meditates will not arrive at a moral framework.

The above passage perfectly captures this sense of ambivalence and the circular reasoning at play: on the one hand, Timothy is indicating that the mindfulness without ethics can be somewhat limited in its efficacy; yet on the other hand, he seems to suggest that meditation (in and of itself) can lead to a moral framework. Moreover, ambivalence about the efficacy of mindfulness was not confined to ethical concerns. A few other participants seemed to harbour doubts as to whether the benefits they gained were resulting from medication or at least a combination of the two treatments (e.g. mindfulness and anti-depressants). Others suggested that faith—whether in some higher power or merely faith in the science of meditation (in case of atheists)—might be a contributing factor in the perceived efficacy of the practice:

Emma [30s; US]: People with faith or some sort of belief and religious leaning of any kind would have more benefit from it. Cause I feel like having that faith could give it more power […] I think that an atheist can find just as much benefit from it too, because if it’s supporting itself, if it’s showing effect in their life, you know they’re gonna say ‘wow it’s a tool and the science behind it makes sense.’

Despite sporadic hits of uncertainty in their narratives (regarding the source of efficacy in mindfulness), the participants commonly reverted to reasoning that if mindfulness is practised regularly, then it will yield positive benefits.

#### The Inner World

Another common theme that emerged involved the conception of mindfulness as a tool for “silencing the mind” and reconnecting with the “inner voice”:

Author: What is the goal of meditation for you?

Basil [50s; UK]: Emptying the mind and not having any thoughts going through my head.

Author: What is the goal of not having any thoughts?

Basil: Being able to listen to that inner voice that speaks to me […]

Author: What is this voice? How is this voice constructed? Is it something that is part of our human nature or is it something that is influenced by our environment and the ideas we absorb from society? Or both?

Basil: Well, I think that the first thing is what definitely speaks to me. In other words, it’s something that we’re all born with. Then, what gets poured into us, what we’re socialised with, that’s the influences of society.

Author: So it changes? Or we’re born with it and...

Basil: No! It’s two separate things. In my view of the world and the way I live it, there is the mind, which is that thing that is full of the stuff that gets put into us by society and by our own experiences. And then there is the other one, which is what we are born with, which is that voice that we cover up with our own minds basically [laughs].

Some participants explained that being in touch with this voice was important because it put them in touch with their moral feelings, thus providing the means to act both rightfully and autonomously.

Rhiannon [40s; US]: It's hard to hear even what your inner voice is trying to say because you're blocking it for so long. So I wanted to see if I could use meditation to help me get in touch with my own, with my intuition and my body and see if I could understand how to work with myself instead of always looking for validation and direction from an outside source.

The assumption expressed by participants is that human beings are born with a moral compass or an intuitive understanding of what is right or wrong. The scope, boundaries, and types of wisdom one might acquire by focusing inwards and accessing the inner voice can, however, surpass the dominion of morality: one can consult the inner voice for aspirations and guidance on one’s career or marriage, as well as finding the “higher self”, the “love within”, or the sense of “oneness” that unites us with the universe. What is highly valued in all these endeavours is the sense of individual authority and autonomy—the fulfilment of finding, on my own terms, what is right for myself, within myself.

The search for an inner voice can be further examined against the backdrop of yet another pervasive discourse among participants: “you are not your thoughts”. Nearly half of our participants explicitly used this or variations of this phrase in their interviews. Some considered it as the most liberating concept they had learnt from mindfulness. However, contrary to traditional Buddhism, while they rendered *some* thoughts as transient and untrue, they did not question the nature of the self and most conceived of an agent, an observer, “a deeper awareness”, an “I”, and a “me” as real rather than illusory.

### Discussion

In this Study 1, we examined the common beliefs in the transformative power of mindfulness through the analysis of in-depth interviews with mindfulness meditators. Our analysis of the participants’ narratives shows that they embrace a range of beliefs about the transformative powers of mindfulness. These included a conceptualisation of compassion that either rested on the notion of cosmic oneness and/or an innate human quality. The conviction that by virtue of unleashing and/or cultivating compassion, mindfulness has the ability to rid the world from violence and inequity. This was exemplified in the unmeasured conviction that if soldiers practised mindfulness, they are bound to quit the army—a point which was accompanied with a sense of passive acceptance of the unfortunate state of the world (e.g. Rhiannon: “dealing with what is”). Finally, the idea of mindfulness as an introspective technology that enables one to silence the mind’s chatter, journey inwards, and access one’s inner voice. These ideas are, for the most part, built upon a series of epistemic claims and metaphysical assumptions about the nature of self and reality—a bricolage of ideas rooted in Romantic sentiments, which later emerged as central beliefs in the New Age thought. These findings reflect the significance of sociocultural trends in shaping peoples’ expectations and understandings of mindfulness meditation. This study therefore extends the call for a contextualised approach to studying mindfulness.

## Study 2

Study 2 comprised the quantitative assessment of people’s beliefs in the powers of mindfulness. To measure people’s beliefs in the transformative powers of mindfulness meditation, we developed a novel scale on beliefs of how this practice can effect change across the personal, interpersonal, and societal domains.

The existing scales of mindfulness are typically designed to measure psychological qualities or traits rather than beliefs. For example, the Five Facet Mindfulness Questionnaire (FFMQ; Baer et al., 2006) includes dimensions such as “acting with awareness”, “nonjudging of”, and “nonreactivity to” inner experience. As far as we are aware, it has never been tested whether these qualities may in some way be related to beliefs one holds about mindfulness meditation. However, we can suppose that people who hold greater beliefs in the transformative powers of mindfulness would tend to rate themselves as having more developed mindfulness qualities. Furthermore, although our scale does not assess level of practise, all statements refer to beliefs about the value of “mindfulness meditation”. Given this, we expected that individuals who have practised for longer and more frequently would have a stronger belief in the powers of mindfulness meditation.

Finally, if we take into account the wider societal framework in which mindfulness arose in the West, and its connection to ideas about Romantic individualism (finding the inner self) and the New Age movement, we expected that individuals scoring high on beliefs in the powers of mindfulness would also score high on self-reported spirituality but not religiosity.

### Method

#### Participants

A total of 458 participants completed our survey. One hundred and fifty-two participants were recruited through online study advertisements asking for non-religious participants with mindfulness meditation practice, primarily Facebook and Twitter (participants received no monetary reward but participated in a raffle for two £50 Amazon vouchers). A total of 306 additional participants were recruited through the online recruitment platform Prolific (these participants were paid £3 for their time). There were 246 female (53.7%), 203 male (44.3%), and 8 other-gender (1.7%) participants. The age of the participants ranged from 18 to 73 years old, with a mean of 35.6 years (*SD* = 12.3). Participants self-identified as atheist (31.7%), followed by agnostic (20.5%), unaffiliated (“no religion”, 18.1%), spiritual but not religious (15.9%), indifferent (3.7%), other (3.9%), Christian (2.8%), Buddhist (2.8%), and Sikh (0.2%). Finally, 225 (49.1%) participants were from the UK, and 233 (50.9%) from the USA.

#### Procedure

Our survey was hosted online on Qualtrics, accessed by participants via a URL. First, participants read a participant information sheet, followed by a consent form. Then it was checked that all participants were indeed individuals who practise mindfulness meditation through the question “Do you practise mindfulness meditation regularly, or have you done so in the recent past?”; those who did not were redirected to the end of the survey. Remaining participants then completed the survey before being thanked for their time. This study was approved by the Ethics Committee of the university.

#### Measures

##### Belief in the Powers of Mindfulness Scale (BPMS)

On the basis of the range of ideas explored under the theme “interpersonal relationships and compassion” of the qualitative assessment, we developed Items 1, 2, and 3; on the basis of the theme “peace and violence”, Items 4 and 5; on the basis of the theme “the inner world”, Items 6 and 7; and together with Items 8 and 9 which capture the desire for self-transformation, we suggest that they make up a comprehensive scale measuring key beliefs about the power of mindfulness meditation to transform the individual and the society. Items were presented in a randomised order to the participants.

For this scale, participants were asked to indicate their agreement with each of the statements, on a Likert scale anchored from *Strongly disagree* (–2.5), to *Disagree* (–1.5), *Somewhat disagree* (–0.5), *Somewhat agree* (0.5), *Agree* (1.5), and *Strongly agree* (2.5). Note that participants were presented with verbal labels only, and the scores are in fractions to maintain even intervals between each Likert option, while also maintaining a neutral zero point. We aimed to recruit a minimum of 300 participants to validate the scale, in line with recommendation for scale development (Comrey & Lee, 2013).

##### Frequency and Length of Mindfulness Meditation

We asked participants how often and how long they had been practising mindfulness meditation for. The options given for length were as follows: *0–6 months*, *6–12 months*, *1–2 years*, *2–5 years*, *5–10 years*, and *More than 10 years*; and the options given for frequency were as follows: *Never*, *Less than once a month*, *Approximately once a month*, *A few times a month*, *A few times a week*, *Once a day, Twice a day*, and *More than twice a day.*

##### Five Facet Mindfulness Questionnaire (FFMQ)

We used the Five Facet Mindfulness Questionnaire (Baer et al., 2006) to assess the participants’ self-reported mindfulness skill. This scale consists of five subscales: Observing, Describing, Acting with awareness, Nonjudging of inner experience, and Nonreactivity to inner experience. Example items of these subscales are (respectively) as follows: “I notice smells and aromas of things”, “I am good at finding words to describe my feelings”, “I find myself doing things without paying attention” (reversed), “I think some of my emotions are bad or inappropriate and I should not feel them” (reversed), and “I perceive my feelings and emotions without having to react to them”. Participants were asked to indicate for each of the scale’s statements whether it is generally true for them, on a Likert scale ranging from *Never or very rarely true* (0), to *Rarely true* (1), *Sometimes true* (2), *Often true* (3), and *Very often or always true* (4). Reliability of the subscales was good, with the following Cronbach alphas and McDonald’s omegas: Observe (*α* = 0.84, ω = 0.84), Describe (*α* = 0.92, ω = 0.87), Act with awareness (*α* = 0.84, *ω* = 0.92), Non-reactivity (*α* = 0.75, ω = 0.84), and Non-judgment (*α* = 0.94, *ω* = 0.94). For the overall scale, Cronbach’s alpha was 0.93 and McDonald’s omega was 0.92. An average score was created for each of the subscales as well as of the whole scale for every participant.

##### Spirituality and Religiosity

We measured self-report spirituality and religiosity through the following two questions: “How spiritual do you rate yourself to be?” and “How religious do you rate yourself to be?” Available options for answers were numbers from 0 to 6 with the following anchors at the extremities: *Not at all* (0) and *Extremely so* (6).

#### Data Analyses

To prepare the data for data analysis, a factor analysis was run to determine the underlying factor of the BPMS, followed by reliability analyses of each scale in the study, after which averages were created for each of the scales. These averages were then used in the correlation analyses, *t*-tests, and ANOVAs, to assess associations between the BPMS and variables of interest, and to compare BPMS scores between relevant groups.

### Results

**Fig. 1:**
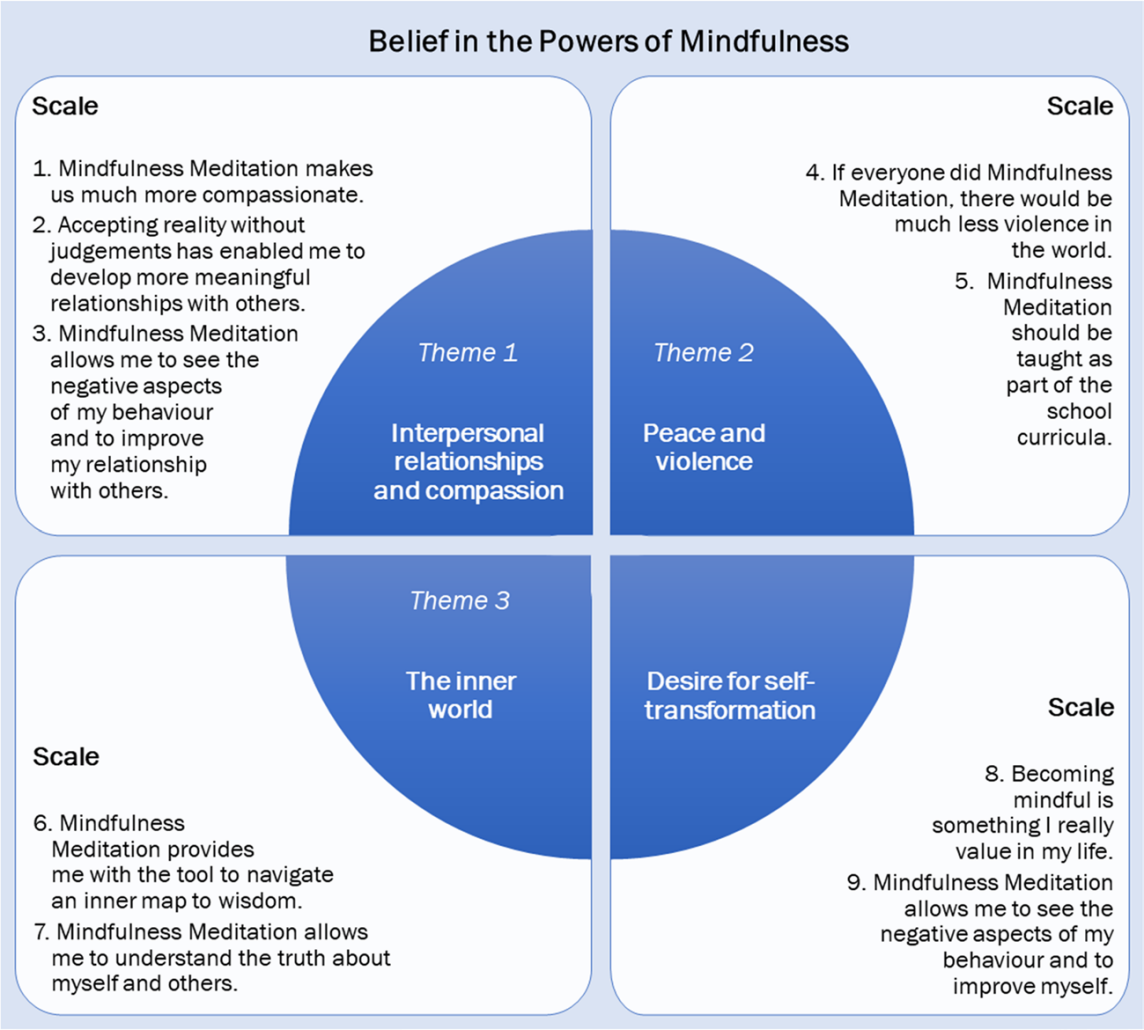
Overview of the BPMS scale items of Study 2 in relation to the qualitative themes and findings of Study 1

Assumptions of normality were assessed for the data, and it was determined that these assumptions were satisfied through statistically significant Shapiro–Wilk tests for the items. Next, a principal axis factor analysis with Varimax rotation (which maximises the variance of the squared loadings within each factor while minimising the cross-loadings between factors) was done on the items of Belief in the Powers of Mindfulness Scale to illuminate the shared variance and to explore the number of factors to be extracted. The first unrotated factor accounted for 54.0% of the common variance (eigenvalue = 4.86). Considering a tapering off in the scree plot, and the fact that the next factor’s eigenvalue was 0.85, accounting for just 9.4% of the common variance, no further factors were analysed. Factor loadings yielded were between 0.60 and 0.74; see Table 1. Both Cronbach’s alpha and McDonald’s omega for these items was 0.89, indicating high reliability and internal consistency, which was not further improved by item elimination. See Fig. 1 for a further overview of these items in relation to the themes.

**Table 1:** Items and factor loadings for the Belief in Powers of Mindfulness Scale (BPMS)

Nr	Item	Factor loading
1	Mindfulness Meditation makes us much more compassionate	0.74
2	Accepting reality without judgements has enabled me to develop more meaningful relationships with others	0.72
3	Mindfulness Meditation allows me to see the negative aspects of my behaviour and to improve my relationship with others.†	0.70
4	If everyone did Mindfulness Meditation, there would be much less violence in the world	0.69
5	Mindfulness Meditation should be taught as part of the school curricula	0.60
6	Mindfulness Meditation provides me with the tool to navigate an inner map to wisdom	0.73
7	Mindfulness Meditation allows me to understand the truth about myself and others	0.73
8	Becoming mindful is something I really value in my life	0.71
9	Mindfulness Meditation allows me to see the negative aspects of my behaviour and to improve myself	0.63

†Given the double-barrelled nature of this item (i.e. two statements within 1 item), we suggest that in future research this item is either split into two or used without the second statement given Item 2; i.e. “Mindfulness Meditation allows me to see the negative aspects of my behaviour”

#### BPMS and Demographic Variables

First, we explored whether demographics—in particular, age, gender, and country of residence—had an effect on belief in the transformative powers of mindfulness meditation. We found that the correlation between the BPMS and age was positive and significant, *r* = 0.14 [0.04, 0.23], *p* = 0.003, indicating that older participants had a stronger belief in the powers of mindfulness meditation. There was no significant difference in scores on the BPMS between women (*M* = 1.63, *SD* = 0.85) and men (*M* = 1.47, *SD* = 0.97), *t*(439) = 1.79, *p* = 0.07. With regard to country of residence, we found that belief in the powers of mindfulness meditation was significantly greater in the USA (*M* = 1.68, *SD* = 0.88) than in the UK (*M* = 1.44, *SD* = 0.92), *t*(447) = –2.87, *p* = 0.004.

#### BPMS and Mindfulness Practice

**Fig. 2:**
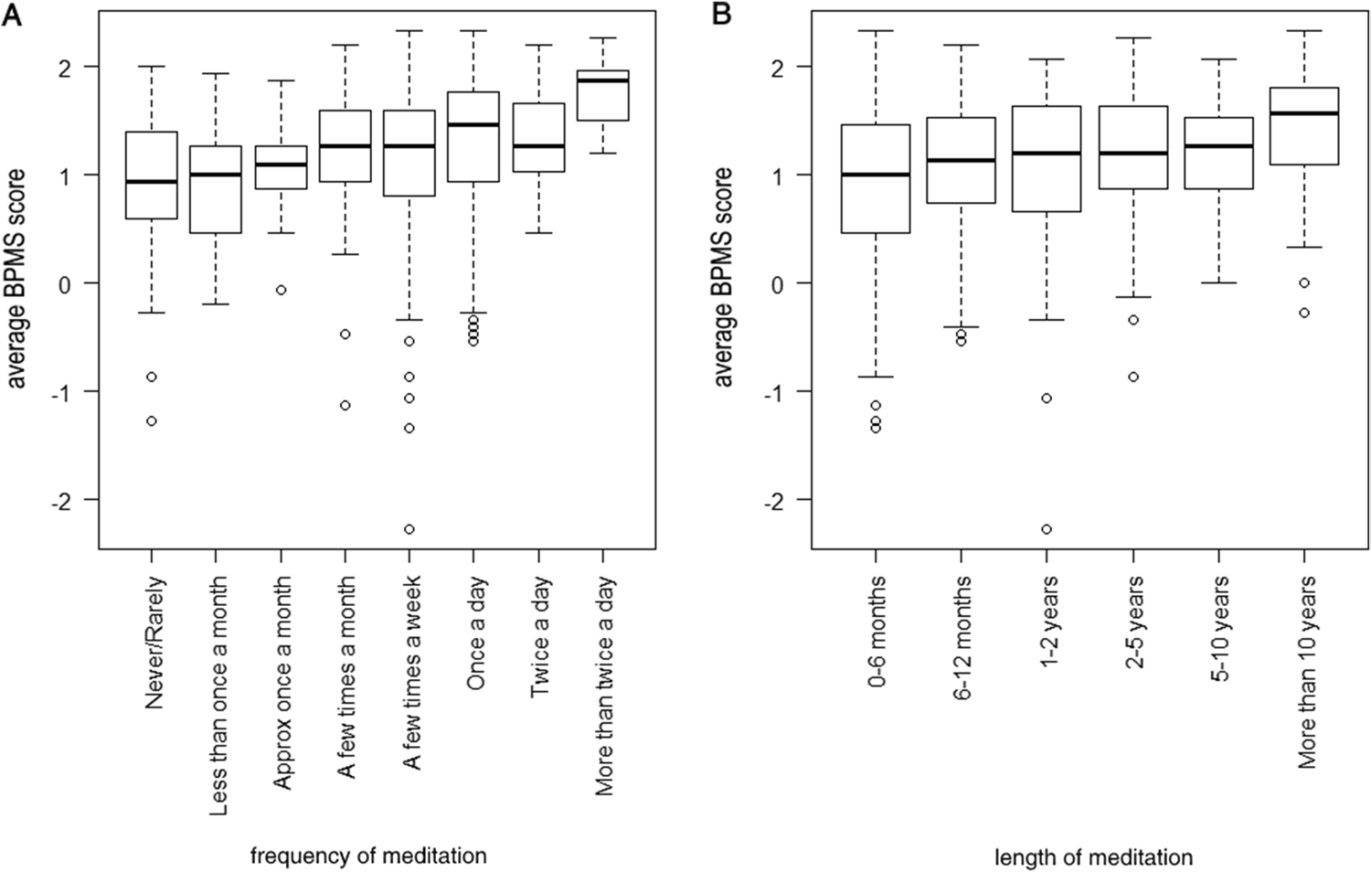
Boxplots showing average belief in mindfulness score by **A** frequency and **B** length of meditation

Next, we examined whether length of time that participants had practised mindfulness meditation and the frequency of their mindfulness meditation practice were associated with increased belief in the powers of mindfulness meditation. We ran ANOVAs on BPMS by length and frequency of mindfulness meditation to test this, and we found that, as expected, participants who had meditated for longer (*F*_(5,446)_ = 7.26, *p* < 0.001, *η*_p_^2^ = 0.07) and participants who meditated more frequently (*F*_(7,444)_ = 8.71, *p* < 0.001, *η*_p_^2^ = 0.12) scored higher on the BPMS; see Fig. 2.

#### BPMS and Self-reported Mindfulness Skill

We predicted that self-report of mindfulness skills would be associated with greater belief in the powers of mindfulness and tested this hypothesis through correlation analyses between BPMS and the subscales of the FFMQ. We found that scores on the BPMS were significantly correlated with scores on all subscales of the FFMQ (with 95% confidence intervals [LL, UL]): Observing, *r* = 0.37** [0.28, 0.44], *p* < 0.001; Describing,* r* = 0.19** [0.11, 0.29], *p* < 0.001; Acting with awareness, *r* = 0.18** [0.10, 0.27], *p* < 0.001; Non-reactivity to inner experience, *r* = 0.38** [0.29, 0.45], *p* < 0.001; Non-judging of inner experience, *r* = 0.12**, [0.05, 0.23], *p* = 0.01.

#### BPMS, Spirituality, and Religiosity

The overall mean score for self-reported spirituality was short of the mid-point but not near 0 (*M* = 2.13, *SD* = 1.65 on a scale from 0 to 7), while the overall mean scores for self-reported religiosity were particularly low (*M* = 0.33, *SD* = 0.71 on a scale from 0 to 7), as expected given the recruitment of non-religious participants. Belief in the powers of mindfulness was associated with greater levels of spirituality (*r* = 0.28 [0.19, 0.37], *p* < 0.001), but not religiosity (*r* = 0.03 [–0.07, 0.12], *p* = 0.62).

### Discussion

In this Study 2, we set out to develop and test our nine-item Belief in the Powers of Mindfulness Scale which overlapped with the themes we found in Study 1 (i.e. interpersonal relationships and compassion, peace and violence, the inner world, and desire for self-transformation). We found that the scale showed good internal consistency and convergent validity. We found, in line with our expectations, that beliefs in the transformative powers of mindfulness were greater in individuals who had meditated for longer and more frequently. We also found that self-reported mindfulness skills were significantly and positively correlated with BPMS. Participants from the USA scored higher on BPMS than those from the UK, as did older participants as compared to younger. Higher levels of spirituality, but not religiosity, were associated with greater beliefs in the transformative powers of mindfulness. This scale will prove useful as quantitative measures in future psychology studies, through its relatively quick assessment of underlying beliefs people have about mindfulness meditation.

## General Discussion

Mindfulness and its potential power in producing individual and societal transformations are heralded by many with great enthusiasm—from meditators to researchers and clinicians. However, little is known about the content and variety of beliefs in the transformative power of mindfulness, in particular in meditators themselves, despite this potentially having important effects on the outcomes of mindfulness meditation. Here we examined these beliefs through a mixed-method approach, which involved longitudinal interviews and a large-scale survey including a newly developed scale on beliefs in the power of mindfulness. We also placed these beliefs in the context of relevant ideologies, validated the new Belief in the Powers of Mindfulness Scale (BPMS), and examined associations of these beliefs with other relevant variables, such as self-reported mindfulness skills.

The Belief in the Powers of Mindfulness Scale consists of items reflecting beliefs relating to the themes of interpersonal relationships and compassion, peace and violence, inner world, and the desire for self-transformation, and had good internal consistency and convergent validity. In a sample of 458 participants from the USA and the UK, we found that individuals who practised mindfulness more often and longer, and were more spiritual, scored higher on this measure. Beliefs in Mindfulness also correlated positively and significantly with the Five Facet Mindfulness Questionnaire (FFMQ), though the effect sizes were medium-sized. This indicates that people who believe in the power of mindfulness also tend to rate themselves higher on mindfulness skills.

So far, mindfulness research has rarely taken beliefs and expectations into account, despite their potential on affecting measurement outcomes. The study by Canby et al. (2021) is an exception to this trend: taking account of social factors, this study observed that instructor- and group-related factors played a significant role in therapeutic trajectories, suggesting that social and environmental conditions may be more important in predicting outcomes than the amount of meditation practise itself (Canby et al., 2021). A neglect of underlying beliefs and expectations is largely due to the assumption that mindfulness meditation is the primary agent of change and that measurable outcomes are exclusively the result of the practise (Canby et al., 2021; Landau et al., 2022). In other words, mindfulness studies have largely privileged a decontextualised approach to mindfulness—examining the technique and its effects in isolation from the larger social and cultural (and indeed the linguistic and historical) contexts in which it is embedded. Here, we contextualised the beliefs in the transformative powers of mindfulness and have created a scale to capture these beliefs for use in future research. We suggest that accounting for participants’ beliefs and expectations—such as through the new Belief in the Powers of Mindfulness scale—may be able, to an extent, to mitigate the shortcomings of self-report measures in clinical trials.

The participants’ key beliefs in the transformative powers of mindfulness were thematically presented in three sections: interpersonal relationships and compassion, peace and violence, and the inner world. As demonstrated in the qualitative section, the participants’ narratives and understanding of the workings of mindfulness were undergirded by a host of ideologies, epistemic claims, and metaphysical assumptions about the nature of mind, self, and reality—which are predicated by broader cultural trends such as expressive individualism, perennial philosophy, and New Age sentiments and ideals.

For instance, the conviction that mindfulness can reduce violence in the world was tightly intertwined with the assumptions that human beings are innately good, that we are born with a “moral compass”, and that mindfulness has the power to cultivate compassion (towards self and others) regardless of how and to whom the practice is taught. The idea that mindfulness has the power to unleash compassion was itself predicated on the conception of compassion as an innate human quality and/or conceptually intertwined with the notion of “cosmic oneness”, which closely mirrored Jon Kabat-Zinn’s articulations of emptiness and interconnectedness (Braun, 2017, p. 192).

Similarly, the conception of mindfulness as a tool for silencing the mind’s chatter and accessing one’s inner voice was grounded on several assumptions: that somewhere within us exists an unconstructed mode of being; that our access to this state is impaired by our mind, which itself is conditioned by our life experiences and our sociocultural, historical, and linguistic contexts; and that by silencing the mind through mindfulness meditation, one could peel back through these layers and connect to a “pure” state that exists distinct from our everyday conscious mind. This conception of the power of mindfulness is, as Robert Sharf (2015) has argued, influenced by a particular take on perennialism called “filter theory”—the idea that our normal sensory and discursive processes filter out reality rather than illuminate it. Interestingly, the filter theory discourse is also popular among some scholars of near-death experience (NDE), such as Bruce Greyson (2021), who propose that NDEs are essentially the “break down” of the brain’s filtering ability: that the cessation of the brain’s ordinary functioning during severe physiological trauma (e.g. clinical death) allows one to tune into a channel otherwise obscured by the mind’s chatter. In other words, that which people experience during an NDEs including out of body experience, meeting dead relatives, and being in the loving presence of a being of light or Jesus are in fact experiences of *unfiltered* reality. The filter theory in the context of NDE studies therefore implicitly supports metaphysical truth-claims about the nature of reality, and the existence of consciousness outside of our physical form (afterlife). In the context of mindfulness, the filter theory discourse rests on similar sets of assumptions about the mind and reality, and the power of the practice rests in its ability to illuminate the bare facets of reality as it is, although the precise nature of reality—that which the mindfulness practitioners conceive as pure or ultimate reality—can significantly vary for each participant.

While the participants in this research framed the initial appeal of mindfulness in terms of its science-based approach to mental health, their thinking about mindfulness is conceptually inherited from various strands of thoughts that are neither scientific (in its epistemology) nor necessarily secular. The analysis of the longitudinal interviews showed that the participants’ ideas and articulation of the purpose of their practise often changed over time, with some taking the form of a spiritual quest for meaning. Indeed, as Study 2 demonstrates, BPMS strongly correlated with self-report spirituality, and all subscales of the Five Facet Mindfulness Questionnaire, as well as length and frequency of practise. Together, these results raise questions about the degree to which perceived benefits are driven by culturally informed beliefs and expectations rather than various claimed mechanisms of action. As detailed above, some of the participants in this study (e.g. Emma, Alfred, Alana) seemed to harbour this ambivalence about the source of efficacy in mindfulness—with some attributing it partially to faith, ethics, or medication.

As shown, the participants in this research conveyed an affinity for a range of ideas that were loaded with metaphysical assumptions. This is significant once we consider the fact that self-identification as non-religious was a criterion for participant recruitment in this research. Although self-identification as non-religious does not necessarily equate to metaphysical-naturalism (the rejection of all supernatural and metaphysical phenomena), one might assume that those who self-categorise as non-religious are less likely to endorse metaphysical claims in comparison to the general public. However, it is also important to note that the ideas about the transformative powers of mindfulness were also shaped by cultural trends—far broader than the perceived religious/secular divide—that have arguably become a general feature of our modern self-understanding (Taylor, 2007).

Our thematic analysis of the interviews shows that while all participants felt that mindfulness meditation positively impacted their interpersonal relationships, they also exhibited opposing ideas about what constitutes a positive behavioural change in this context (e.g. willing to engage with other’s opinion vs. walking away/disengage). This demonstrates that people’s interpretations were highly subjective; and the behavioural patterns that they considered as prosocial effects of mindfulness may not objectively appear as such. To put simply: one person’s notion of exercising self-compassion in a context of an interpersonal conflict can be construed as self-centric and antisocial from another’s perspective. This finding should come as a cautionary tale for scholars who primarily rely on self-report measures to assess the impact of mindfulness on prosocial behaviour. For instance, this poses a challenge to Bihari and Mullan’s (2014,, p. 53) grounded theory model, “relating mindfully”, which depicts an uncomplicated representation of the changes in relationships brought about by mindfulness. Our finding therefore problematises the existing methodologies (e.g. self-report measures) involved in studies—and by extension, meta-analyses—that claim mindfulness enhances prosocial behaviour (Donald et al., 2019), and illustrates the persisting importance of attending to people’s beliefs and expectations in future studies/trials (Greenberg et al., 2006).

### Limitations and Future Research Directions

There are some limitations to this research. In our studies, we recruited individuals who self-identified as non-religious (atheist, agnostic, nones). While some evidence indicates that non-religion may be a popular stance among mindfulness meditators in Western context (Landau et al., 2022) and in countries such as Vietnam where majority of the population indicate no religious affiliation (Tran et al., 2022), there may be issues with generalisability for other contexts. We also recruited participants who were approached primarily through online platforms, such as social media (Facebook and Twitter) and a participant recruitment platform (Prolific). This may have introduced potential biases in participant recruitment, as individuals who use these platforms may have different characteristics compared to those who do not. Indeed, those who volunteered through social media platforms on average practised mindfulness more often and for longer, suggesting some self-selection through the snowball sampling on social media, and participation for monetary incentives on Prolific. However, participants were also recruited in-person during fieldwork within several mindfulness communities. Moreover, we were interested in mindfulness practitioners of all levels and with varying interests, the achievement of which was helped by recruiting participants through a number of different channels. Another limitation to the Belief in the Powers of Mindfulness scale is that we used a double-barrelled item: “Mindfulness Meditation allows me to see the negative aspects of my behaviour and to improve my relationship with others”. This is generally discouraged in scale development as participants may be responding more to the first statement or to the second statement, or indeed are not able to reconcile the two statements. Therefore, we have added the recommendation to the factor analysis overview that future researchers use just the first statement of this item, “Mindfulness Meditation allows me to see the negative aspects of my behaviour”, given that Item 2 already covers the belief in mindfulness meditation as a way to improve one’s relations: “Accepting reality without judgements has enabled me to develop more meaningful relationships with others.”

A final limitation of the scale is that it may have been impacted by social desirability bias, where participants answer the survey in a way that reflects well on themselves, or that are in line with the popularity and popular narrative of mindfulness, rather than disclosing their true thoughts. However, the anonymous responses to the survey (Study 2) were in line with the interviews of Study 1, indicating that the aspect of anonymity had little effect on the results. The convergence of answers in Study 1 and Study 2 despite the different methods of extracting information about the participants’ beliefs (i.e. survey versus interviews) further validates these findings. Moreover, the scale includes a number of items that go beyond the popular narrative (e.g. “Mindfulness Meditation should be taught as part of the school curricula”). Together, this suggests a minimal effect of social desirability bias.

For future research, we suggest that researchers should consider the specific understandings that participants have regarding mindfulness, as this may significantly direct their attitudes and behaviours. We envision that our scale, the BPMS, will be useful in measuring people’s expectations in mindfulness intervention trials as well as helping understand the extent to which such beliefs play an important role in the everyday lives of mindfulness practitioners.

## Data Availability

The qualitative data in Study 1 cannot be publicly shared because the participants did not give written consent for their interview transcripts to be shared publicly. The quantitative data in Study 2 are available from the corresponding author, MR, upon reasonable request.
